# Causes of Disease

**Published:** 1866-08

**Authors:** 


### CAUSES OF DISEASE.

"I THANK you very much for the valuable counsel and improved health you have given me. I feel confident that most of the diseases to which clergymen are subject arise from their own imprudence, or perhaps ignorance. We attribute many of our ailings to the visitation of God's Providence, when we had better call them the visitations of our own folly. We pray for vigor and strength of body when we pursue a course of conduct which sets all the known and unknown laws of health at defiance. I believe that a vast amount of disease, save that which is hereditary, is as much the fault of the patient as delirium tremens is the fault of the drunkard. I like your JOURNAL and its motto. You 'labor for the good time coming,' you will die before it comes; but your words, your principles, and influence will work on, in unforgotten power. I feel so much better, I see no reason why I should not say perfectly well. I labor and study with delight—think I am the happiest man alive."

It may add to the interest and value of this case, to observe further, that the writer was the efficient minister of an influential people—was on the point of giving up his charge, either permanently, or for a tour to the continent of Europe; but writing for our advice, we encouraged him to hold on, work hard, and get well, under the circumstances under which he expected to remain. The result of a month's treatment tells its own story. This is a good advertisement of our skill thus far, but we choose to tell the whole story. We thought he was more scared than hurt, rather more desponding than the circumstances of the case warranted; for beyond a pill or two—whether of bread, assafœtida, or solidified aqua fortis, the deponent sayeth not—we sent him nothing but some good advice, which, in divers similar cases, did no good at all; the difference being simply this, he had intelligence, self-denial, and decision. He had sense enough to be instructed as to the nature of his case, to appreciate the adaptation of the means proposed, and the moral courage to compel himself to the observance of those means; hence he staid at home, stood his ground, worked hard, and got well. In our branch of medicine, we never could cure a soul, not a single soul, of that class of persons who know every thing and more too. To get well of any chronic disease, of a serious character, and to remain cured, a man must be led to see the nature of his own case, the needs and requirements of his own constitution, and must have that force of character which compels compliance with those requisitions. As long as the world stands, the ignoramus and the animal will die before his time. Intelligent self-denial is the price of health and long life the world over: it never will be otherwise.

